# Evaluation of triblock copolymeric micelles of δ- valerolactone and poly (ethylene glycol) as a competent vector for doxorubicin delivery against cancer

**DOI:** 10.1186/1477-3155-9-42

**Published:** 2011-09-25

**Authors:** Lekha Nair K, Sankar Jagadeeshan, S Asha Nair, G S Vinod Kumar

**Affiliations:** 1 Chemical Biology, Molecular Medicine Division, Rajiv Gandhi Centre for Biotechnology, Poojappura, Thiruvananthapuram-695 014, Kerala, India; 2 Cancer Research, Rajiv Gandhi Centre for Biotechnology, Poojappura, Thiruvananthapuram-695 014, Kerala, India

## Abstract

**Background:**

Specific properties of amphiphilic copolymeric micelles like small size, stability, biodegradability and prolonged biodistribution have projected them as promising vectors for drug delivery. To evaluate the potential of δ-valerolactone based micelles as carriers for drug delivery, a novel triblock amphiphilic copolymer poly(δ-valerolactone)/poly(ethylene glycol)/poly(δ-valerolactone) (VEV) was synthesized and characterized using IR, NMR, GPC, DTA and TGA. To evaluate VEV as a carrier for drug delivery, doxorubicin (DOX) entrapped VEV micelles (VEVDMs) were prepared and analyzed for *in vitro *antitumor activity.

**Results:**

VEV copolymer was successfully synthesized by ring opening polymerization and the stable core shell structure of VEV micelles with a low critical micelle concentration was confirmed by proton NMR and fluorescence based method. Doxorubicin entrapped micelles (VEVDMs) prepared using a modified single emulsion method were obtained with a mean diameter of 90 nm and high encapsulation efficiency showing a pH dependent sustained doxorubicin release. Biological evaluation in breast adenocarcinoma (MCF7) and glioblastoma (U87MG) cells by flow cytometry showed 2-3 folds increase in cellular uptake of VEVDMs than free DOX. Block copolymer micelles without DOX were non cytotoxic in both the cell lines. As evaluated by the IC_50 _values VEVDMs induced 77.8, 71.2, 81.2% more cytotoxicity in MCF7 cells and 40.8, 72.6, 76% more cytotoxicity in U87MG cells than pristine DOX after 24, 48, 72 h treatment, respectively. Moreover, VEVDMs induced enhanced apoptosis than free DOX as indicated by higher shift in Annexin V-FITC fluorescence and better intensity of cleaved PARP. Even though, further studies are required to prove the efficacy of this formulation *in vivo *the comparable G2/M phase arrest induced by VEVDMs at half the concentration of free DOX confirmed the better antitumor efficacy of VEVDMs *in vitro*.

**Conclusions:**

Our studies clearly indicate that VEVDMs possess great therapeutic potential for long-term tumor suppression. Furthermore, our results launch VEV as a promising nanocarrier for an effective controlled drug delivery in cancer chemotherapy.

## Background

In spite of the current advances in cancer, chemotherapy still faces the major problem of lack of selectivity of anticancer drugs towards neoplastic cells [1]. The efficacy of chemotherapy is decided by maximum tumor cell killing effect during the tumor growth phase and minimum exposure of healthy cells to the cytotoxic agent. Continuous and steady infusion of the drug into the tumor interstitium is also desirable to exterminate the proliferating cells, to finally cause tumor regression. Advances in nanotechnology have resulted in the evolution of a variety of nano-sized carriers for controlled and targeted delivery of chemotherapeutics [2-4]. Moreover, recent advances in polymer based micelles have opened new frontiers for drug delivery [5,6] and tumor targeting [7].

Amphiphilic block copolymers have the tendency to self-assemble into micelles in a selective solvent because of the presence of both, hydrophilic as well as hydrophobic segments [8,9]. These polymeric micelles consist of a core and shell like structure, in which the inner core is the hydrophobic part and can be utilized for encapsulation of drugs, whereas the hydrophilic block constituting the outer shell provides stabilization. The potential of polymeric micelles as drug carriers lie in their unique properties like small size, prolonged circulation, biodegradability and thermodynamic stability [10,11]. Moreover, these micelles have the ability to preferentially target tumor tissues by enhanced permeability and retention effect due to the small size of the carrier molecule which facilitates the entry within biological constraints proving their superiority over other particulate carriers [12,13]. Another important characteristic of these micelles is the presence of water compatible polymers like polyethylene glycol (PEG) which improves the bioavailability of these drug delivery systems [14,15]. PEG not only saturates these polymeric particles with water by making them soluble, but also prevents opsonization of these nanocarriers by providing steric stabilization against undesirable aggregation and non-specific electrostatic interactions with the surroundings [16,17]. This has resulted in an extensive study of drug formulations using copolymeric micelles with enhanced antitumor efficacy [18-20]. Although, a number of polyester based copolymers like caprolactone, valerolactone and lactides have been studied [21-23], serious investigations on δ-valerolactone based copolymeric micelles for drug delivery applications are scarcely reported in literature. For example, doxorubicin based copolymeric micelles have been investigated [24,25], but the potential of δ-valerolactone and PEG based micelles as carriers for controlled delivery is yet to be explored. Doxorubicin (DOX), an anthracycline antibiotic, is a drug used in the treatment of a large spectrum of cancers especially breast, ovarian, brain and lung cancers [26]. However, its therapeutic potential is limited due to its short half life [27] and severe toxicity to healthy tissues resulting in myelosuppression and cardiac failure [28,29].

Hence, the aim of this work was to use a δ-valerolactone based amphiphilic block copolymer to develop a novel micellar controlled delivery system for DOX and analysis of its anticancer activity. The present study involves the synthesis of a triblock copolymer of δ-valerolactone, poly δ-valerolactone)/poly(ethylene glycol)/poly(δ-valerolactone) (VEV) by ring opening polymerization and characterization using IR, NMR and GPC. The thermal stability of VEV was analyzed using DTA and TGA. Micellization followed by biocompatibility studies of the copolymer were done to evaluate its potential as a carrier for drug delivery. DOX entrapped VEV micelles (VEVDMs) were prepared and characterized using TEM and the *in vitro *release kinetics at two different pH. Their biological evaluation was done in two different cancer cell lines, breast adenocarcinoma (MCF7) and glioblastoma (U87MG). Cellular uptake of micelles was observed and compared to free DOX using confocal microscopy and FACS. Furthermore, the antiproliferative activity was analyzed by MTT assay, Annexin V-FITC staining and western blot analysis followed by alterations in cell division cycle.

## Results

### Synthesis and characterization of triblock copolymer

**Figure 1:**
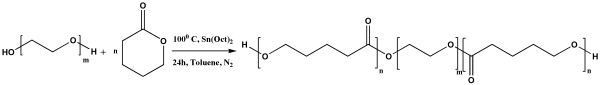
**Scheme of polymer synthesis**. Synthetic schematic diagram of synthesis of Poly(δ-valerolactone)/Poly(ethylene glycol)/Poly(δ-valerolactone) (VEV) copolymer using δ-valerolactone and poly(ethylene glycol) as monomers by ring opening polymerization using stannous octoate as a catalyst is represented.

The synthetic pathway for the synthesis of VEV is shown in Figure 1. Ring opening polymerization technique using stannous octoate was implemented to synthesize triblock amphiphilic copolymer of δ-valerolactone using PEG_2000_.

**Figure 2:**
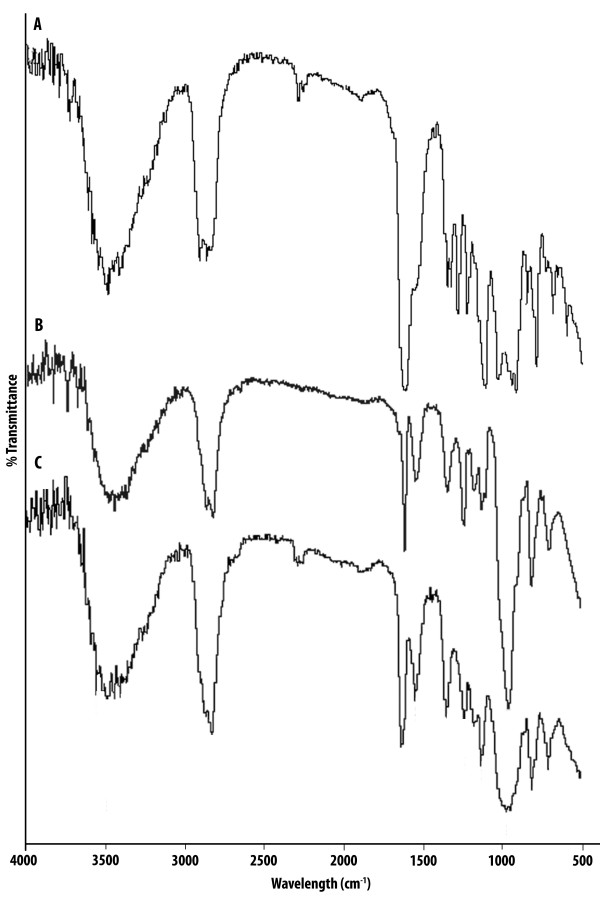
**Fourier Transform Infra Red spectra**. FT-IR spectra of commercially bought monomers (A) δ-Valerolactone (VL) (B) Poly(ethylene glycol) (PEG) and the synthesized copolymer VEV (C) Poly(δ-valerolactone)/Poly(ethylene glycol)/Poly(δ-valerolactone) (VEV) copolymer were recorded using potassium bromide pellets.

The chemical structure of obtained VEV copolymer was confirmed using FT-IR and ^1^H NMR. In the FT-IR spectra of the copolymer, the characteristic bands at 2875 cm^-1 ^and 1100 cm^-1 ^represent the C-H stretching and C-O-C band of PEG, respectively. The band at 1726 cm^-1 ^attributed the carbonyl (-C = O) stretching of the δ-valerolactone monomer, respectively (Figure 2).

**Figure 3:**
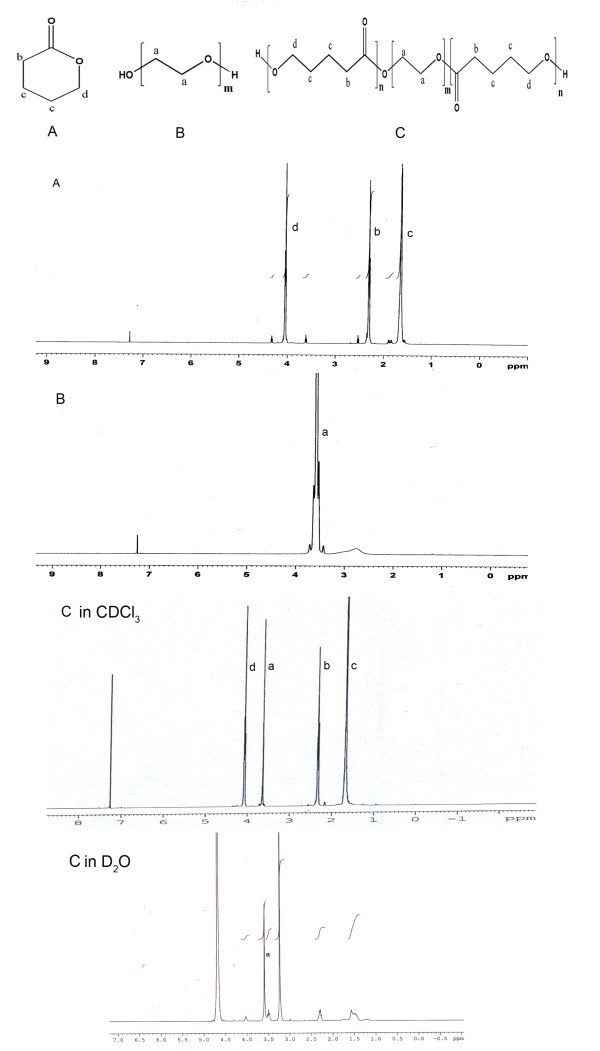
**^1^H Nuclear Magnetic Resonance spectra**. ^1^H NMR spectra of commercially bought monomers (A) δ-Valerolactone (VL) (B) Poly(ethylene glycol) (PEG) and the synthesized copolymer VEV (C) Poly(δ-valerolactone)/Poly(ethylene glycol)/Poly(δ-valerolactone) (VEV) copolymer were recorded in CDCl_3 _and D_2_O as solvents.

The ^1^H NMR spectra acquired in deuterated chloroform, which is a good solvent for both blocks, contained signals from the protons of PEG as well as PVL. The chemical shifts at ~3.6 ppm (4H, H_a_) indicated the -CH_2 _protons of PEG whereas the characteristic chemical shifts of δ-valerolactone were seen at 2.4 ppm (2H, H_b_), 1.6 ppm (4H, H_c_) and 4 ppm (2H, H_d_) as shown in Figure 3, confirming the successful synthesis of VEV copolymer [30].

**Figure 4:**
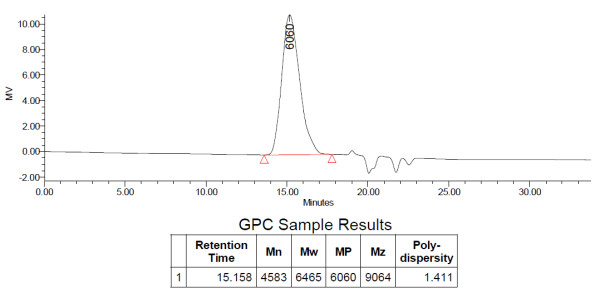
**Molecular weight distribution**. The molecular weight of the synthesized VEV copolymer was determined using gel permeation chromatography (GPC) on a liquid chromatography system using tetrahydrofuran (THF) as the eluent.

The molecular weights and single peak with a narrow molecular weight distribution in the GPC chromatogram of the synthesized VEV copolymer suggests the efficiency of polymerization (Figure 4).

**Figure 5:**
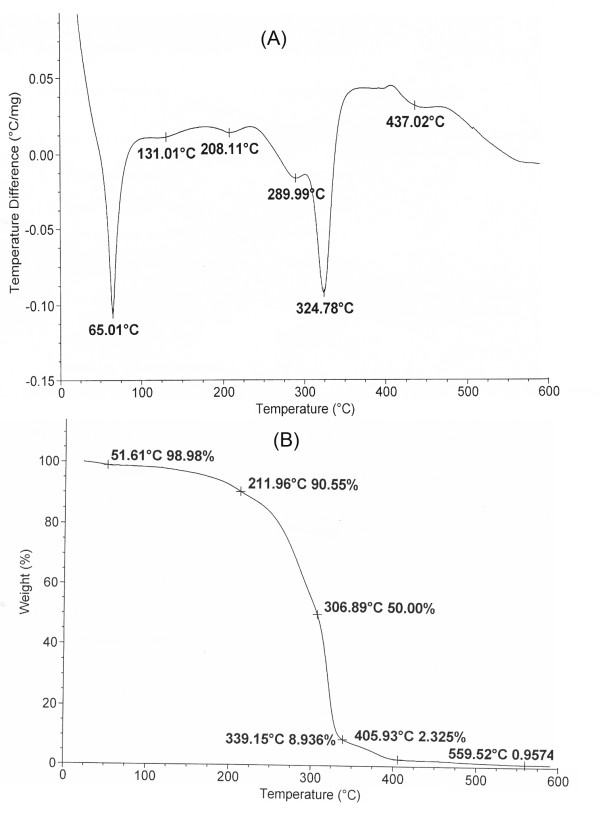
**Thermal analysis of VEV copolymer**. (A) Differential thermal analysis (DTA) and (B) Thermogravimetric analysis (TGA) of VEV copolymer were recorded under nitrogen flow at a scanning rate of 10°C min^-1^.

Furthermore, thermal analysis of VEV showed a melting point near 65.01°C (Figure 5A) which is higher than that of the individual monomers and thermodynamic stability up to a temperature of 211.9°C indicating the increase in stability on polymerization (Figure 5B).

### Micellization and characterization

Since VEV is an amphiphilic copolymer, it is expected to form a core-shell type micelle structure in aqueous media. NMR analysis showed protons of both VL and PEG on using CDCl_3 _as a solvent. However, with D_2_O clear signals of only PEG blocks were seen (Figure 3) which suggests that PVL due to its hydrophobicity forms the inner core whereas PEG is the exposed hydrated corona. VEV copolymeric micelles were characterized using particle size analyzer for their size and polydispersity. As shown in Table 1, copolymer VEV gave micelles in nanometer range with a low polydispersity. Also the low CMC value for micelle formation suggests that VEV can be a good nanocarrier for drug delivery.

**Table 1:** Characterization of VEV micelles

Polymer	Micelle size (nm)	PDI^a^	CMC^b ^(mg/L)
VEV	83 ± 2.5	0.17 ± 0.008	1.16 ± 0.03

^a^Polydispersity

^b^Critical micelle concentration

### Preparation and properties of DOX loaded copolymeric micelles (VEVDMs)

**Figure 6:**
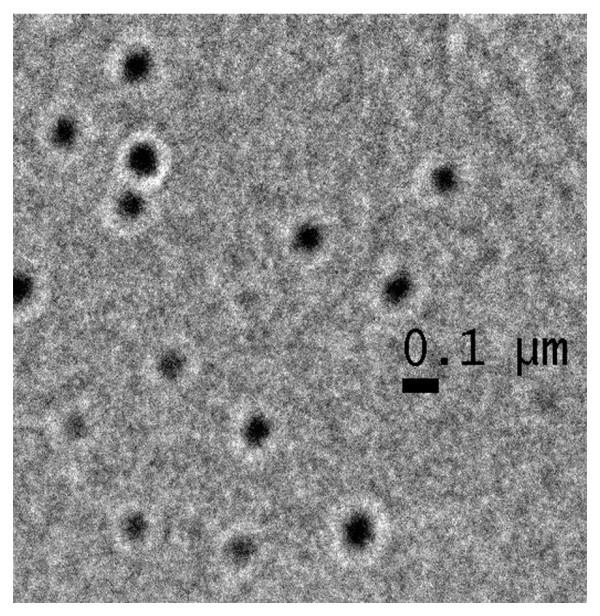
**Transmission Electron Microscope image of doxorubicin loaded VEV micelles (VEVDMs)**. For TEM, the sample of VEVDMs suspension in water milli-Q was dropped onto formvar-coated grids without being negatively stained. Measurements were taken only after the sample was completely dried.

Avoiding the time consuming and low encapsulation efficiency yielding methods like dialysis and nanoprecipitation [25], we employed a novel single emulsion method for the preparation of DOX loaded copolymeric micelles using copolymer VEV. In spite of aqueous solubility of DOX the modified single emulsion method yielded micelles in the size range of 90 nm (Figure 6) with high drug entrapment efficiency and yield (Table 2).

**Table 2:** Characterization of doxorubicin loaded VEV micelles (VEVDMs)

Sample	Encapsulation efficiency%	Diameter (nm)	Yield%	Polydispersity
VEVDMs	56.2 ± 2.4	90.4 ± 3.5	80.9 ± 4.0	0.173 ± 0.01

**Figure 7:**
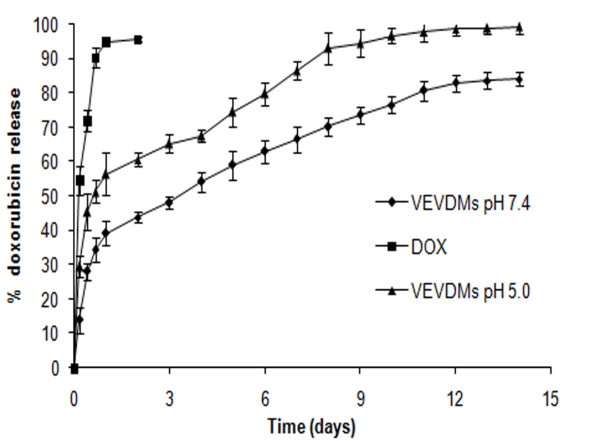
***In vitro *release of doxorubicin from micelles**. Release pattern of free doxorubicin in comparison to DOX entrapped in VEV micelles in phosphate buffer at pH 7.4 and pH 5.0, and 37°C. All the measurements were done in triplicate. The results are expressed as arithmetic mean ± standard error on the mean (S.E.M).

Stability studies of VEVDMs showed that there was no significant change in micelle mean size and polydispersity index upon storage at 4°C for a period of one year (data no shown). Also, VEVDMs were easily redispersible in water which is very important for their application in drug delivery. The drug release profile from DOX loaded micelles showed that VEVDMs were able to sustain DOX release for more than two weeks with dependence on the pH of the release media (Figure 7). VEVDMs at pH 7.4 released only 15% DOX in the first hour whereas almost double amount of DOX was released at pH 5 during the same time. At pH 5, almost 100% DOX was released in two weeks but at pH 7.4 more than 15% of drug remained entrapped. However, free DOX at pH 7.4 and 5, diffused quickly through the dialysis membrane with almost 90% release with in 24 h. These results indicate that DOX release from VEVDMs is controlled and pH dependent.

### VEVDMs showed enhanced cellular uptake

**Figure 8:**
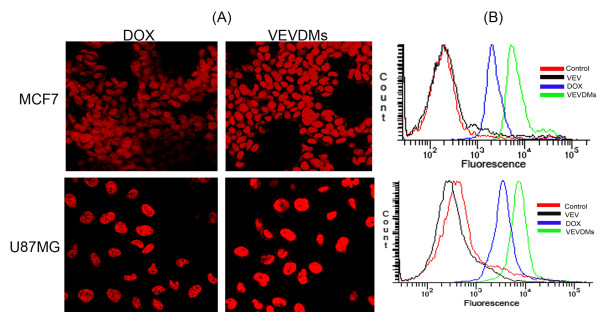
**Sub-cellular internalization of DOX entrapped VEV micelles (VEVDMs)**. MCF7 and U87MG cells were treated with 1 μM DOX formulations. Micelle uptake of VEVDMs by MCF7 and U87MG cells in comparison to free DOX after 2 h of incubation at 37°C is shown by (A) CLSM images showing the internal fluorescence of DOX in cells at a magnification of 60× (B) Comparison of fluorescence intensity by flow cytometry to analyze the extent of internalization.

To analyze the cell uptake of VEVDMs by MCF7 and U87MG cells, intracellular fluorescence of DOX was evaluated using CLSM and the fluorescence intensity of micelles was compared to free DOX using FACS. Confocal images showed better intensity of fluorescence in both the cell lines when incubated with VEVDMs in comparison to its free state. For a quantitative analysis of intracellular uptake, the fluorescence intensity in cells incubated with DOX formulations was compared using flow cytometer. It is worth noting here that the intensity of MCF7 cells and U87MG cells incubated with VEVDMs showed almost 2-3 folds increase in cellular uptake in comparison to free DOX (Figure 8).

### Micellar DOX of non-toxic VEV copolymer exhibited better in vitro cytotoxicity with smaller IC_50 _values

**Figure 9:**
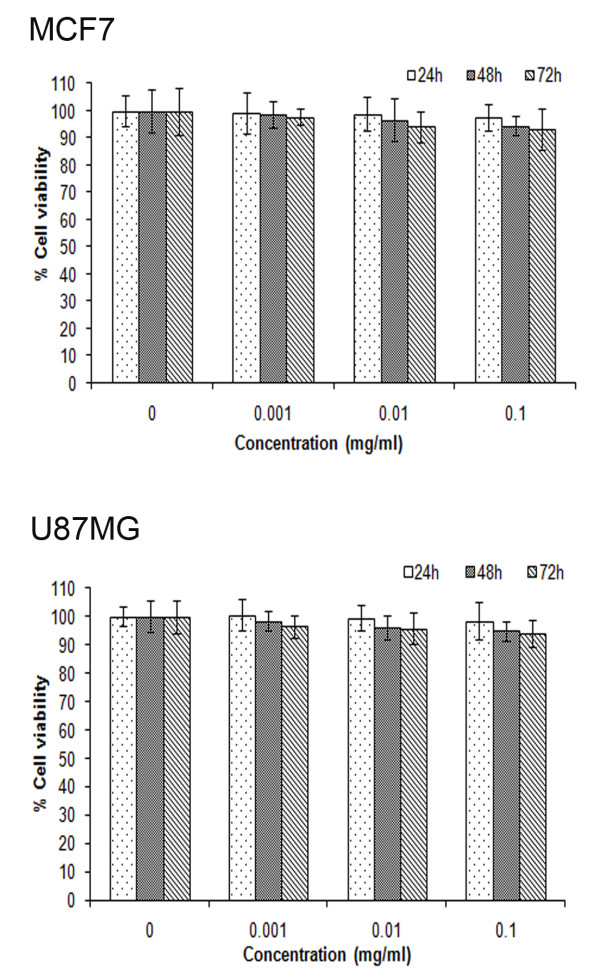
**Cytotoxicity study of VEV copolymer**. The biocompatibility analysis of empty VEV micelles on MCF7 and U87MG cells at 24, 48 and 72 h on incubation with the concentrations as indicated was analyzed using MTT assay. All the measurements were done in six replicates. The results are expressed as arithmetic mean ± standard error on the mean (S.E.M).

Before analyzing VEV micelles as carriers for drug delivery we checked its cytotoxicity in MCF7 and U87MG cell lines. The cells were exposed to varying concentrations of VEV ranging from 0.001 mg/ml to 0.1 mg/ml for 24, 48 and 72 h and checked for cytotoxicity. VEV triblock copolymeric micelles showed no cytotoxicity to highest copolymer concentration (0.1 mg/ml) tested even after 72 h incubation (Figure 9). This suggests that neither VEV nor its hydrolysis products are toxic showing the ability of VEV to be used as a carrier for drug delivery.

**Figure 10:**
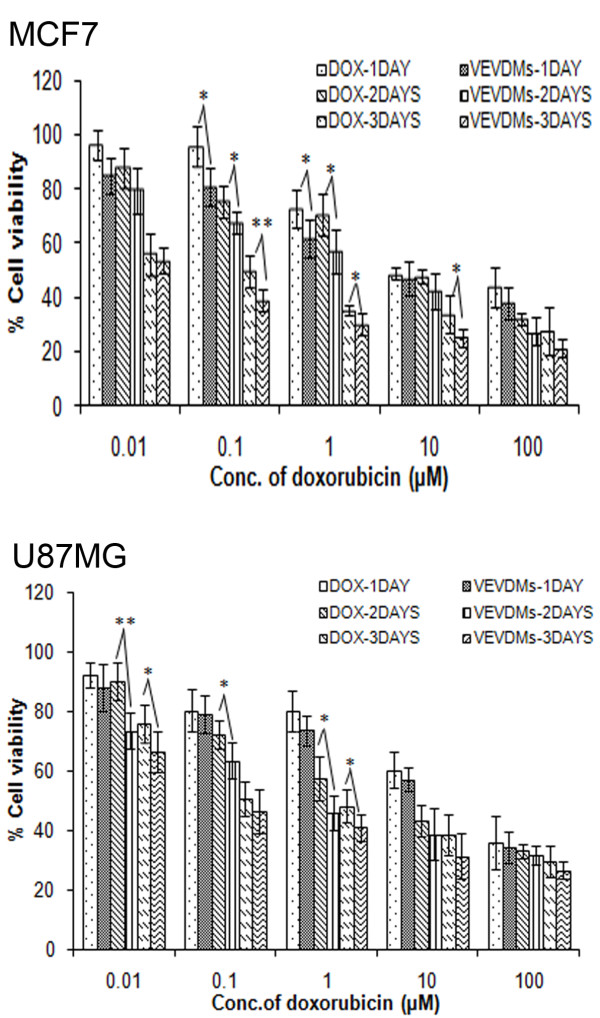
**Cell viability assay**. Comparison of the cell viabilities of MCF7 and U87MG cells on treatment with free DOX and equivalent concentrations of VEVDMs as indicated on 24, 48 and 72 h incubation was done by MTT. All the measurements were done in six replicates and the results are expressed as arithmetic mean ± standard error on the mean (S.E.M) with statistical significance *p < 0.05, **p < 0.01.

The cytotoxicity of free DOX and VEVDMs with increasing concentrations of 0.01-100 μM was evaluated in both the cell lines for 24, 48 and 72 h using MTT assay. VEVDMs exhibited enhanced cytotoxicity to both the cells when compared to pristine DOX in a dose and time dependent manner (Figure 10). The IC_50 _values calculated from dose responsive curve summarized in Table 3 showed that VEVDMs gave much lower IC_50 _values than pristine DOX at all the time durations showing that micellar DOX was more potent in killing cancer cells.

**Table 3:** IC_50 _values (in equivalent μM DOX) of MCF7 and U87MG cells cultured with VEVDMs vs. free doxorubicin in 24, 48, 72 h

Incubation time (h)	IC_50 _MCF7 cells (μM)		IC_50 _U87MG cells (μM)	
	**Free DOX**	**DOX micelles**	**Free DOX**	**DOX micelles**

24	25.8	5.73	31.21	18.471
48	8.239	2.369	4.149	1.138
72	0.05	0.0091	0.7255	0.1517

### Annexin V-FITC showed enhanced apoptosis by VEVDMs

**Figure 11:**
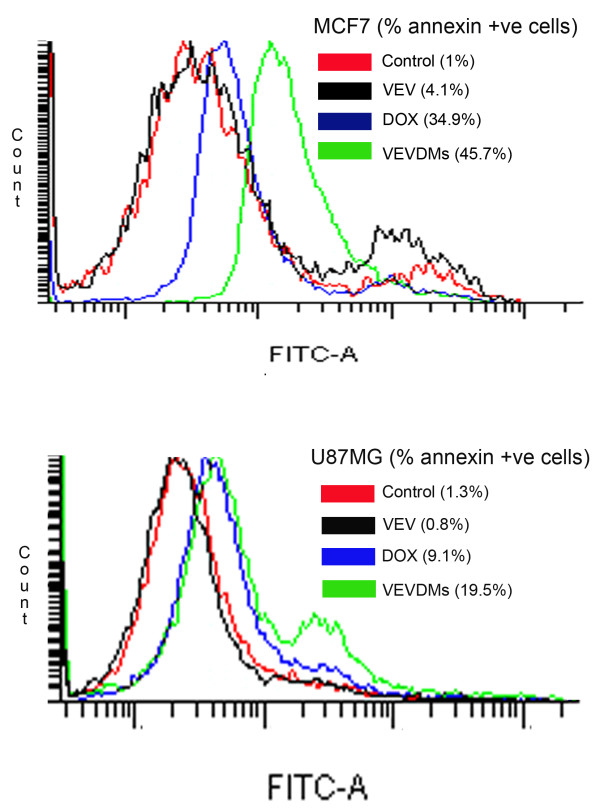
**Apoptosis analysis by FACS using Annexin V-FITC stain assay**. MCF7 and U87MG cells were incubated with 1 μM of DOX formulations for 24 h. To compare apoptosis, FITC-conjugated annexin binding to phosphatidyl serine, exposed to the outer leaflet, on treatment with DOX formulations was measured by FACS.

To measure and compare the extent of apoptosis induced by 1 μM of free DOX and VEVDMs on incubation for 24 h, FITC-conjugated annexin staining was done and analyzed using flow cytometer. Annexin staining which identifies cell surface changes that occur in the early stages of apoptosis show a right shift in the FACS histogram due to fluorescence emitted by apoptotic cells. The histogram of VEVDMs treated cells on annexin staining suggested that 45.7 and 19.5% MCF7 and U87MG cells underwent apoptosis, whereas only 34.9 and 9.1%, MCF7 and U87MG cells were apoptotic after treatment with equivalent concentration of free DOX. Also, empty VEV micelles showed no annexin shift like that of untreated control which indicates its biocompatibility (Figure 11).

### Better PARP cleavage induced by VEVDMs

**Figure 12:**
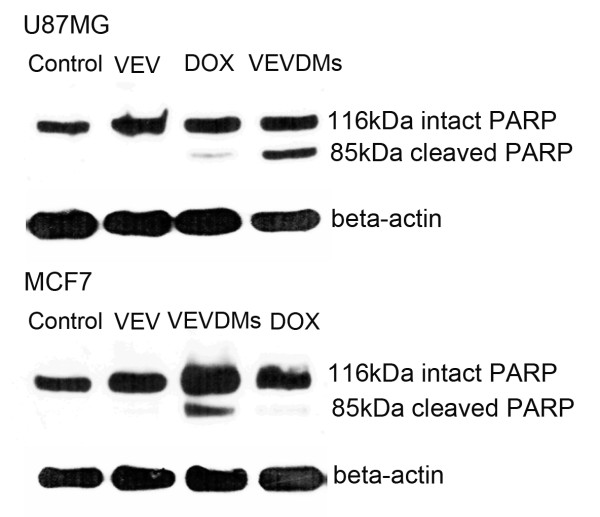
**PARP cleavage determination by western blot analysis**. Comparison of PARP cleavage induced by 3 μM of VEVDMs to free DOX in MCF7 and U87MG cells on 24 h incubation. Immunoblotting was carried out using antibodies specific for PARP and detected using enhanced chemiluminescence method.

To detect the cleavage of PARP, a DNA repairing protein and hallmark of apoptosis, western blot was done. Immunoblot analysis showed that the intensity of the 116-kDa PARP decreased considerably in both the cell lines on incubation with DOX micelles in comparison to the groups treated with the same concentration of free DOX (Figure 12). Since PARP cleavage is a clear indicator of apoptosis, these results show the efficiency of VEVDMs to cause cell death.

### Induction of cell cycle arrest by low concentrations of VEVDMs

**Figure 13:**
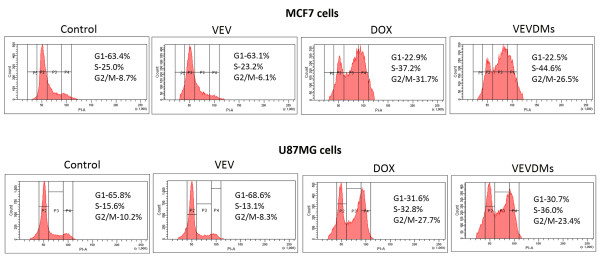
**Cell cycle arrest analysis by FACS**. Effect of 0.05 μM VEVDMs treatment on cell cycle of MCF7 and U87MG cell lines in comparison to a double concentration of 0.1 μM free DOX on 24 h incubation was assessed by FACS.

Since same concentration of DOX micelles showed better results of cytotoxicity and apoptosis, we analyzed the influence of VEVDMs on cell cycle at a concentration half that of free DOX using flow cytometer. As DOX is known to induce G2/M phase arrest, the cells treated with DOX formulations showed a clear G2/M arrest. However, it is important to note that both MCF7 and U87MG cells (Figure 13) showed a comparable G2/M phase arrest accompanied by a significant S phase arrest with VEVDMs even at concentration half that of free drug which clearly indicates their superior activity.

## Discussion

**Figure 14:**
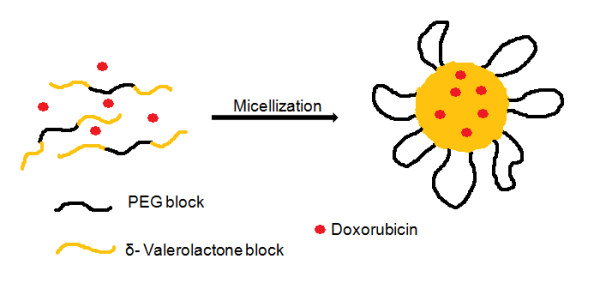
**Schematic illustration to represent the structure of VEVDMs**. Schematic diagram showing the structure of micelles formed on DOX entrapment in VEV copolymeric micelles. The micelle is represented by a hydrophobic PVL core and hydrophilic PEG on the surface with the drug entrapped inside the hydrophobic matrix.

Polymeric micelles using triblock copolymers have been widely studied for drug delivery due to their properties that include thermodynamic stability, increased bioavailability, enhanced solubilization of poorly soluble drugs and targeting ability [5]. Although, numbers of copolymers based on PEG have been already reported, the real potential of δ-valerolactone based triblock copolymer is poorly addressed. In the present study we report the synthesis, characterization and *in vitro *antitumor evaluation of δ-valerolactone and PEG based triblock copolymeric micelles for the delivery of anticancer agent, doxorubicin. Effective ring opening polymerization using stannous octoate was carried out using δ-valerolactone with PEG having molecular weight of 2000 (Figure 1). Confirmation of the synthesis of new copolymer poly(δ-valerolactone)/poly(ethylene glycol)/poly(δ-valerolactone) (VEV) was done using IR (Figure 2) and NMR (Figure 3). In agreement with the previous reports, good polymerization efficiency with low PDI values (Figure 4) was obtained with the selected low molecular weight of PEG, PEG_2000 _[31]. One of the major reasons behind studying δ-valerolactone based micelles for drug delivery was that the thermodynamic as well as kinetic stability of micelles is expected to increase with the increase in the hydrophobicity and state of the micelle core [31]. VEV showed good thermal stability (Figure 5) which is in agreement with previous reports [32] and may be attributed to the high hydrophobic nature of δ-valerolactone. VEV formed stable micelles (Table 1) having inner PVL core and outer PEG blocks (Figure 14) as explained from NMR studies and is because of its amphiphilic nature [30].

These micelles were further assessed for biological evaluation of VEV as a carrier using doxorubicin (DOX) as the model drug.

The modified single emulsion solvent evaporation method adopted for the preparation of DOX loaded VEV micelles (VEVDMs) not only proved to be simple and efficient for the fabrication of drug entrapped micelles but also gave particles in the size range of 90 nm (Figure 6) with high encapsulation efficiency and yield (Table 2). Here, the particle size is a very important physical parameter because it directly affects the cellular uptake capability. The analysis of DOX release from micelles showed a biphasic pattern with first phase of slight burst release followed by second phase of sustained release continuing over a period of two weeks (Figure 7). The drug release from micelles showed pH dependence which might be due to the variation in the hydrolysis of ester chain and DOX solubility with changing pH [33,34]. This slow and sustained release from VEVDMs could be more desirable for the delivery of DOX to solid tumors *in vivo*. Although, actual application need the evaluation of these micelles in animal models, sustained drug release from VEVDMs supports the idea of using VEV copolymer based micelles for controlled delivery of anticancer agents.

Enhanced intracellular uptake of VEVDMs by MCF7 and U87MG cells as shown by confocal images and FACS (Figure 8) may be attributed to the small size of drug loaded micelles with PEG on their surface. Since few studies have reported that based on biocompatibility ε-caprolactone based copolymers are better for drug delivery applications in comparison to δ-valerolactone [15,17], we analyzed the cytotoxicity of VEV and found that the copolymer showed no cytotoxicity at concentrations up to 0.1 mg/ml even on incubation of 3 days (Figure 9). Since lesser concentrations of drug loaded micelles are for administration, no issues of biocompatibility are expected. Moreover, after dilution with large volume of body fluid *in vivo*, 0.1 mg/ml represents a much higher intravenous material dose than required for *in vivo *drug delivery. Therefore, VEV can be considered to be non toxic and biocompatible. However, intracellular toxicity evaluation of VEVDMs induced higher cell killing in both cells in a concentration and time dependent manner (Figure 10). Considerable lower IC_50 _values of VEVDMs (Table 3) might be due to the enhanced cellular uptake accompanied by a slight burst release which showed acceleration in acidic conditions. Furthermore, higher shift in Annexin V-FITC fluorescence (Figure 11) and intensity of cleaved PARP (Figure 12) which are clear indicators of apoptosis as reported in our earlier studies [35], confirms that VEVDMs are more effective in inducing cell death. In agreement with previous reports of DOX induced DNA damage occurring predominantly in the G2/M phase of cell cycle [36], VEVDMs even at half the concentration of free DOX induced comparable G2/M arrest accompanied by a higher S phase arrest (Figure 13). Since cell cycle arrest is doxorubicin concentration and exposure time dependent with higher concentrations inducing delayed S phase transit [37,38], higher S phase arrest by half dose of VEVDMs may serve the same purpose as done by a double amount of free DOX. Thus, the higher apoptosis induced by VEVDMs might also be attributed to the fate of cell cycle arrest. Another very important thing to be noted is that VEVDMs showed better antitumor activity against both the cell lines irrespective of their nature [39], which suggests their use against a variety of tumors.

Hence, our study clearly indicates the challenging potential of VEVDMs for cancer treatment with enhanced micellar stability imparted by the high hydrophobic nature of δ-valerolactone. The superior antitumor efficacy may be accounted on the basis of higher cellular uptake, DOX release in acidic conditions and cell cycle arrest. Although, further evaluation of VEVDMs in *vivo *model is required, these PEGylated micelles certainly possess the tendency to accumulate in solid tumors with increased bioavailability [40,41] to deliver the anticancer agent for a long-lasting tumor containment.

## Conclusions

A novel δ-valerolactone and PEG based triblock copolymer was synthesized and characterized for drug delivery applications. VEV prepared by ring opening polymerization showed good micelle formation tendency and no cellular toxicity. To evaluate VEV as a carrier, DOX was successfully loaded in VEV using a modified single emulsion method with high encapsulation efficiency and yield. DOX release from VEVDMs continued for more than two weeks and was found to be pH dependent. VEVDMs obtained in the size range of 90 nm showed enhanced cellular uptake efficiency and much lower IC_50 _values in comparison to pristine DOX. Moreover, the efficiency of DOX micelles to induce apoptosis accompanied by significant cell cycle arrest supports the idea of using VEVDMs against malignancy. Although additional studies are required to evaluate the *in vivo *behavior of VEV, our results confirm the potential of VEVDMs in chemotherapy and VEV as a carrier for future applications in drug delivery.

## Methods

### Materials

δ-valerolactone (VL), doxorubicin hydrochloride (DOX), stannous octoate, 3-(4, 5-dimethylthiazol-2-yl)-2, 5-diphenyltetrazolium bromide (MTT), Pluronic F-68, Ribonuclease A, 1,6-diphenyl-1,3,5-hexatriene (DPH) and Annexin V apoptosis detection kit were purchased from Sigma-Aldrich, Steinheim, Germany. Polyethylene Glycol 2000 (PEG) was obtained from Merck Schuchardt OHG, Germany. Poly (ADP-ribose) polymerase (PARP) was bought from cell signaling and enhanced chemiluminescence kit from GE Amersham. Human breast adenocarcinoma (MCF7) and glioblastoma (U87MG) cells were provided from ATCC (USA) and maintained in DMEM medium containing 10% fetal bovine serum (Sigma, USA) and 1% antibiotic antimycotic cocktail (Himedia, India). All solvents were of analytical grade.

### Synthesis of poly (δ-valerolactone)/poly (ethylene glycol)/poly (δ-valerolactone) (VEV) copolymer

The triblock copolymer, VEV was synthesized by ring opening polymerization of PEG and VL in the presence of stannous octoate as catalyst as reported [30], with some modifications. In a typical procedure, PEG and VL monomers (molar ratio 1:200) along with stannous octoate (0.005 mol %) were added to the reaction vessel and placed under nitrogen in an oil bath at 110°C with magnetic stirring for 24 h. The resulting mixture was cooled to room temperature, dissolved in dichloromethane and precipitated in an excess amount of cold ether to remove residual monomers. Purification of the copolymer was achieved by the dissolution/precipitation method with dichloromethane and ether, followed by filtration and drying in vacuum.

### Characterization of VEV copolymer

To characterize VEV, Fourier transform infrared (FT-IR) spectra were measured by FT-IR spectrometer (Nicolet 5700) using potassium bromide (KBr) pellets. Proton nuclear magnetic resonance ^1^H spectra (NMR) were obtained using Bruker 500 MHz with deuterated chloroform (CDCl_3_) or water (D_2_O) as solvent. To analyze the molecular weight, gel permeation chromatography (GPC) measurements were carried out on a Waters 515 liquid chromatography system equipped with two Waters Styragel HR 5ETHF columns and a Waters 2414 refractive index detector using tetrahydrofuran (THF) as the eluent (1.0 ml/min). In addition, thermal stability of the polymer was measured by differential thermal analysis (DTA) and thermogravimetric analysis (TGA) using SDT-2960, TA Instruments Inc under nitrogen flow at a scanning rate of 10°C min^-1^.

### Micellization and characterization

VEV polymeric micelles were prepared by a known precipitation method [42]. Briefly, 100 mg of polymer dissolved in 10 ml of acetone was added to 50 ml aqueous media and stirred overnight at room temperature to remove the organic solvent. The polymeric micelles were then lyophilized and resuspended before every analysis. The size of micelles was measured using a particle size analyzer (Beckman Coulter Delsa Nano Particle Analyzer). The critical micelle concentration (CMC) of VEV micelles was determined by fluorescence based method using DPH as a probe [30]. In brief, VEV aqueous solutions were added to DPH solution (0.4 mM), such that the final concentration of copolymer ranged from 0.001-1 wt%. The samples were equilibrated overnight at room temperature and UV absorption was recorded at 365 nm on a UV-VIS spectrophotometer (Perkin Elmer, USA). The critical micelle concentration (CMC) was calculated on the basis of absorption vs. logarithmic polymer concentrations. Micelles were also analyzed by ^1^H NMR using deuterated water as a solvent.

### Preparation and characterization of DOX loaded VEV micelles (VEVDMs)

In a novel method for preparing drug entrapped micelles using a triblock copolymer, single emulsion solvent evaporation method was adopted. Briefly, DOX (1:100 w/w) dissolved in methanol (1:10 v/v) was added to VEV solution in acetone to form the organic phase, which on addition to an aqueous phase containing Pluronic F-68 (1%) gave emulsion containing micelles. This emulsion after sonication was subjected to overnight stirring at room temperature to get micellar suspension. VEVDMs in dry powder form were obtained after centrifugation followed by lyophilization.

Size analysis of VEVDMs was done using particle size analyzer (Beckman Coulter Delsa Nano Particle Analyzer) and images were taken using a transmission electron microscope (TEM, JEOL 1011, Japan). To calculate the drug content in micelles, weighed amount of dried drug loaded micelles were dissolved in DMSO (dimethyl sulphoxide) and the drug amount was calculated according to a standard curve obtained using DMSO solutions of known concentrations of free DOX by UV spectrophotometer (Perkin Elmer, USA) at the detection wavelength 480 nm. The encapsulation efficiency was expressed as the ratio of DOX in micelles to the initial amount of drug used. The yield corresponds to the ratio of amount of micelles recovered to the total amount of polymer and drug used in formulation.

### In vitro drug release

DOX loaded micelles were dispersed in distilled water (1 mg/ml) and then placed in a dialysis bag (M_W _cut off: 3500). The dialysis bag was then immersed in 15 ml of buffer solutions with pH 5.0 (acidic) and 7.4 (physiological) and incubated at 37°C. At specific time intervals, the drug released solution was replaced with equal amount of fresh media and the amount of DOX released was analyzed using UV-vis spectrophotometer at 480 nm. The release kinetics at two different was compared to that of free DOX.

### Cellular uptake studies

To visualize the cellular uptake of drug-loaded micelles, cells were grown on cover slips placed in 24 well plates. After 24 hours cells were treated with 1 μM DOX formulations and incubated for 2 h. The cells were washed, mounted and examined under a confocal laser scanning microscope (CLSM, Leica DMI 4000B) at a magnification of 60× for intracellular DOX fluorescence. Furthermore, the intensity of DOX fluorescence on cellular uptake was analyzed using flow cytometry (FACS Aria, BD, USA). Cells seeded in six-well culture plates (5 × 10^4^) after 24 h incubation, were treated with 1 μM DOX formulations. After exposure of 2 h, the cells were washed with cold PBS three times, harvested using trypsin-EDTA and analyzed for internal fluorescence of DOX using flow cytometer.

### Cytotoxicity studies

To assess the cytotoxicity of empty VEV micelles, free DOX and VEVDMs, 3-(4, 5-dimethylthiazol-2-yl)-2,5-diphenyltetrazolium bromide (MTT) reduction assay was performed [35]. Briefly, human breast adenocarcinoma (MCF7) and gliomablastoma (U87MG) cells were seeded (5.0 × 10^3^/well) and incubated for 24 h in 96-well plates. Cells were incubated with various concentrations of VEV, free DOX and VEVDMs as indicated and incubated for 24, 48 and 72 h, respectively. Following treatment the amount of formazan crystals formed was measured after 4 h of MTT addition (10% v/v) by adding isopropyl alcohol and OD measurement at 570 nm. The relative cell viability in percentage was calculated as (A_test_/A_control_) × 100.

### Annexin V-FITC staining

To examine cell apoptosis induced by DOX formulations, Annexin V-FITC stain assay was performed on both the cell lines [35]. Briefly, cells cultured with or without drug (1 μM) for 24 h were washed in cold PBS and resuspended in binding buffer. Afterwards, cells were stained with FITC-labeled annexin using Annexin V-FITC Apoptosis Detection Kit according to the manufacturer's instructions and a flow cytometric analysis was then carried out using FACS Aria (Special order system, BD, USA).

### Western blot analysis

2 × 10^6 ^cells were seeded in 100-mm culture plates and treatments containing 3 μM DOX was given for 24 h. Cells were then lysed and the total protein content was measured using Bradford's reagent. 50 μg of total protein was loaded for SDS-PAGE. Immunoblotting was carried out using antibodies specific for PARP and detected using enhanced chemiluminescence (ECL) method [35].

### Cell cycle analysis

For flow cytometric analysis, 10^6 ^cells were seeded in six-well culture dishes and given treatment of 0.05 μM VEVDMs and 0.1 μM free DOX for 24 h. Cells were harvested and fixed with 70% ethanol for 1 h. The fixed cells were then given RNAse A (100 mg/ml) treatment for 1 h at 37°C followed by propidium iodide (10 mg/ml) incubation for 15 min. Finally, cells were analyzed using FACS Aria (Special order system, BD, USA) [35].

### Statistics

All the measurements were done in three or more replicates. The results are expressed as arithmetic mean ± standard error on the mean (S.E.M). For cytotoxicity experiments the normalization of the data was done by considering the mean value of the untreated samples as 100%. All other data points were expressed as percentage of the control. Statistical difference (*p < 0.05, **p < 0.01) were calculated using GraphPad Instat 3.

## Competing interests

The authors declare that they have no competing interests.

## Authors' contributions

LNK synthesized and characterized the polymer and nanoparticles and wrote the final manuscript. SJ guided LNK in performing all the biological assays. SAN participated in evaluation of the biological experiments and supplied information for writing the final manuscript. GSVK planned the whole work and corrected the manuscript. All authors read and approved the final manuscript.
